# Case Report: Child with Menkes syndrome complicated by bladder diverticula

**DOI:** 10.3389/fped.2025.1571582

**Published:** 2025-07-04

**Authors:** Guoxing Wu, Pengfei Gao, Wenbin Zhang, Honghui Li, Zhaoying Li, Ruifa Wu, Zuoqing Li, Mingchuan Huang, Zhe Xu

**Affiliations:** ^1^ Department of Urology and Urodynamics Center, Dongguan Children's Hospital, Dongguan, China; ^2^ Department of Pediatric Surgery, The First Affiliated Hospital, Sun Yat-sen University, Guangzhou, China

**Keywords:** Menkes syndrome, bladder diverticula, diverticula excision, urodynamics, prognosis

## Abstract

**Background:**

Menkes syndrome is a rare X-linked genetic disorder of copper metabolism caused by variants in the *ATP7A* gene. It is characterized by developmental delay, hair abnormalities, hypotonia, and organ dysfunction. Bladder diverticula are a rare but recognized urological complication, and its rupture can lead to severe clinical consequences.

**Methods:**

We report a case of a 3-year-old boy diagnosed with Menkes syndrome, presenting with multiple bladder diverticula and diverticular rupture, resulting in acute abdominal effusion. The patient underwent excision of multiple bladder diverticula guided by imaging and urodynamic evaluation. Postoperative functional recovery was assessed through follow-up imaging and urodynamic studies.

**Results:**

Postoperative urodynamic parameters showed significant improvement. Follow-up revealed satisfactory voiding function without evidence of recurrence or increased residual urine. Imaging and urodynamic studies were instrumental in both preoperative localization and postoperative functional assessment.

**Conclusions:**

Early diagnosis and surgical excision of bladder diverticula in patients with Menkes syndrome can significantly improve prognosis. Imaging and urodynamic studies provide reliable support for comprehensive management and are invaluable for long-term postoperative follow-up.

## Introduction

Menkes syndrome is an extremely rare congenital disorder of copper metabolism caused by variants in the *ATP7A* gene and follows an X-linked recessive inheritance pattern (1). Its prevalence is estimated at approximately 1 in 100,000 live births, primarily affecting male infants (2). Clinical manifestations typically appear within the first few months of life and include neurodevelopmental delay, hair abnormalities, hypotonia, seizures, and multisystem involvement (3, 4). Despite advances in understanding its pathophysiology and treatment, the overall prognosis for Menkes syndrome remains poor, with most affected children succumbing to severe complications by the age of three.

Urological complications in Menkes syndrome are common, with bladder diverticulum being particularly common (5). This condition is hypothesized to result from structural weakness of the bladder wall due to connective tissue abnormalities. Rupture of a bladder diverticulum can lead to severe abdominal infections and functional impairment, underscoring the importance of early intervention (6). Currently, no standardized guidelines exist for managing bladder diverticulum in Menkes syndrome, and treatment decisions must consider the patient's life expectancy, disease burden, and family expectations (7).

Here, we report a rare case of a child with Menkes syndrome presenting with multiple bladder diverticula and rupture. The case highlights the role of imaging and urodynamic studies in guiding evaluation and follow-up. Multidisciplinary treatment discussions facilitated individualized treatment, including surgical excision of the bladder diverticula, which yielded favorable clinical outcomes (8). This case contributes valuable insights into the comprehensive management of Menkes syndrome, and underscores the critical role of imaging and urodynamic evaluations in both preoperative planning and postoperative monitoring.

## Case report

### Patient information and medical history

The patient, a 3-year-old boy, was admitted to our department for recurrent abdominal distension and reduced urine output. At the age of 1 year, genetic testing using peripheral blood samples confirmed the diagnosis of Menkes disease, revealing a hemizygous missense variant in the ATP7A gene, NM_000052.7:c.3032C>A (p.Ala1011Asp), located at ChrX:78029365 (based on the GRCh38 reference genome). This variant was classified as likely pathogenic according to ACMG criteria. Trio analysis showed that the variant was *de novo*, with both parents carrying the wild-type allele. At approximately 2 years of age, the patient began receiving subcutaneous histidine-bound copper (copper-histidine) injections, administered once daily at a dose of 1 ml. This therapy was continued through the follow-up period. The child had a history of recurrent infections and developmental delay, but no prior significant urological abnormalities had been identified during evaluations at other hospitals.

### Present illness

One week prior, the patient experienced intermittent fever (moderate grade) for three days and had 6–8 episodes of watery diarrhea daily. Ultrasound at a local hospital suggested “abnormal hypoechoic regions outside the bladder wall, likely representing multiple bladder diverticula, with ascites”. Three days before admission, he developed abdominal distension and reduced urine output without apparent triggers, accompanied by poor appetite and lethargy. Worsening symptoms prompted referral to our department for further management.

### Physical examination and laboratory findings

On admission, the patient appeared ill, with a markedly distended abdomen exhibiting a rounded, tense appearance. Physical examination revealed positive shifting dullness and fluid wave, suggestive of ascites. Bowel sounds were reduced (2 per minute), and bilateral renal tenderness was absent. Laboratory tests revealed elevated serum urea (9.18 mmol/L) and creatinine (54.0 μmol/L), while other parameters, including complete blood count, liver function, electrolytes, and coagulation profile, were within normal limits.

### Imaging and urodynamic evaluation

**Figure 1:**
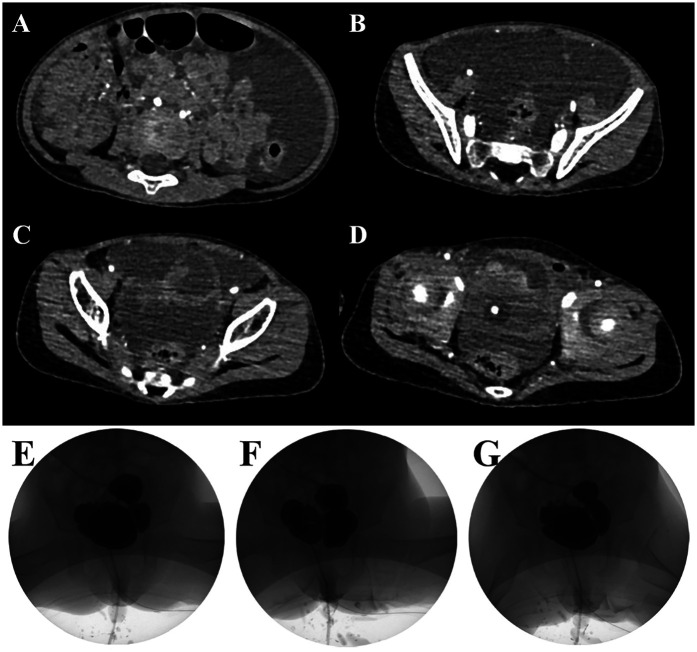
Preoperative abdominal CT scans demonstrating multiple bladder diverticula and ascites in the patient **(A–D)**. Preoperative video urodynamic study showing the bladder filling phase **(E)**, voiding phase **(F)**, and post-void residual phase **(G)**

Preoperative abdominal CT imaging revealed significant ascites and multiple bladder diverticula, the largest measuring approximately 27 × 29 mm, consistent with diverticular rupture (see Figures 1A–D). Although specific bladder wall thickness was not reported, multiple cystic structures were noted adjacent to the bladder. Video urodynamic evaluation performed prior to surgery showed reduced bladder compliance (13 ml/cm H₂O) and a post-void residual urine volume of 54 ml, indicating impaired bladder emptying, which was consistent with urine reflux into the diverticula. The detrusor leak point pressure could not be elicited, and the end-filling detrusor pressure was measured at 5 cm H₂O. Although bladder sensation was preserved, bladder capacity was decreased. Coordinated activity between the detrusor muscle and urethral sphincter was not clearly observed, suggesting possible dysfunction. These findings were in line with the presence of multiple bladder diverticula and a clinically significant degree of lower urinary tract dysfunction. The images during the bladder filling period, the urination period and after urination are shown in Figures 1E–G.

### Initial management

Initial management included urinary catheterization and ultrasound-guided peritoneal drainage, which yielded a large volume of pale-yellow fluid. Post-procedure ultrasound confirmed resolution of ascites, and the peritoneal drain was subsequently removed.

### Surgical management

**Figure 2:**
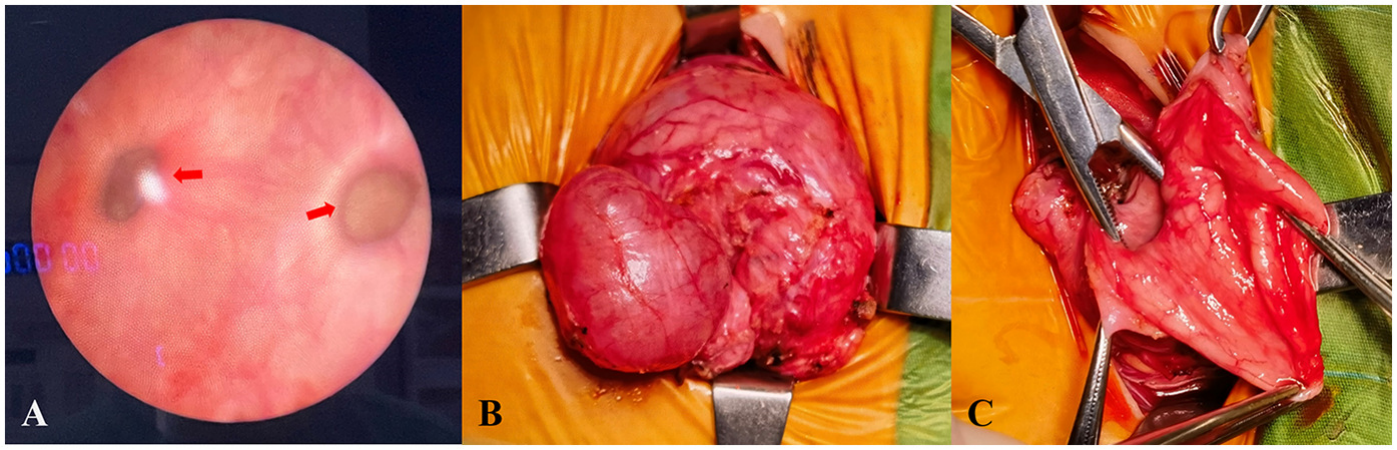
Intraoperative cystoscopy identifying multiple bladder diverticula **(A)** intraoperative images showing surgical excision of the diverticula **(B–C)**.

**Figure 3:**
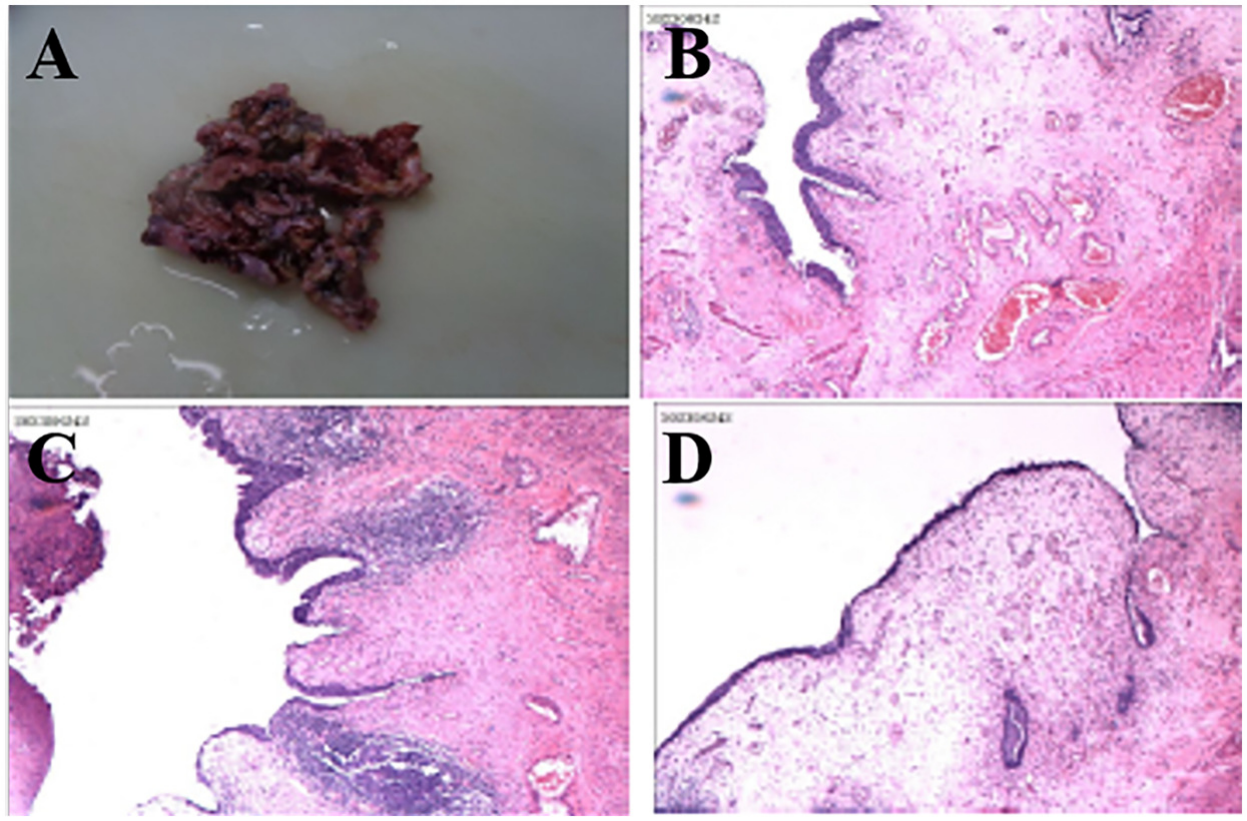
Histopathological analysis confirming diverticular wall degeneration and structural abnormalities, including thinning of the muscularis propria and mucosal changes **(A–D)**.

Following discussion, the patient underwent open surgical excision of multiple bladder diverticula via an extravesical approach. Intraoperatively, several markedly enlarged bladder diverticula with thin walls were identified and completely excised (see Figure 2). No intraoperative complications occurred. Histopathological analysis confirmed diverticular degeneration and structural abnormalities (see Figure 3). Postoperative recovery was uneventful, with successful catheter removal and resumption of normal voiding. Serial postoperative ultrasound assessments revealed no evidence of residual or recurrent diverticula during follow-up (see “Follow-Up”).

### Follow-Up

**Figure 4:**
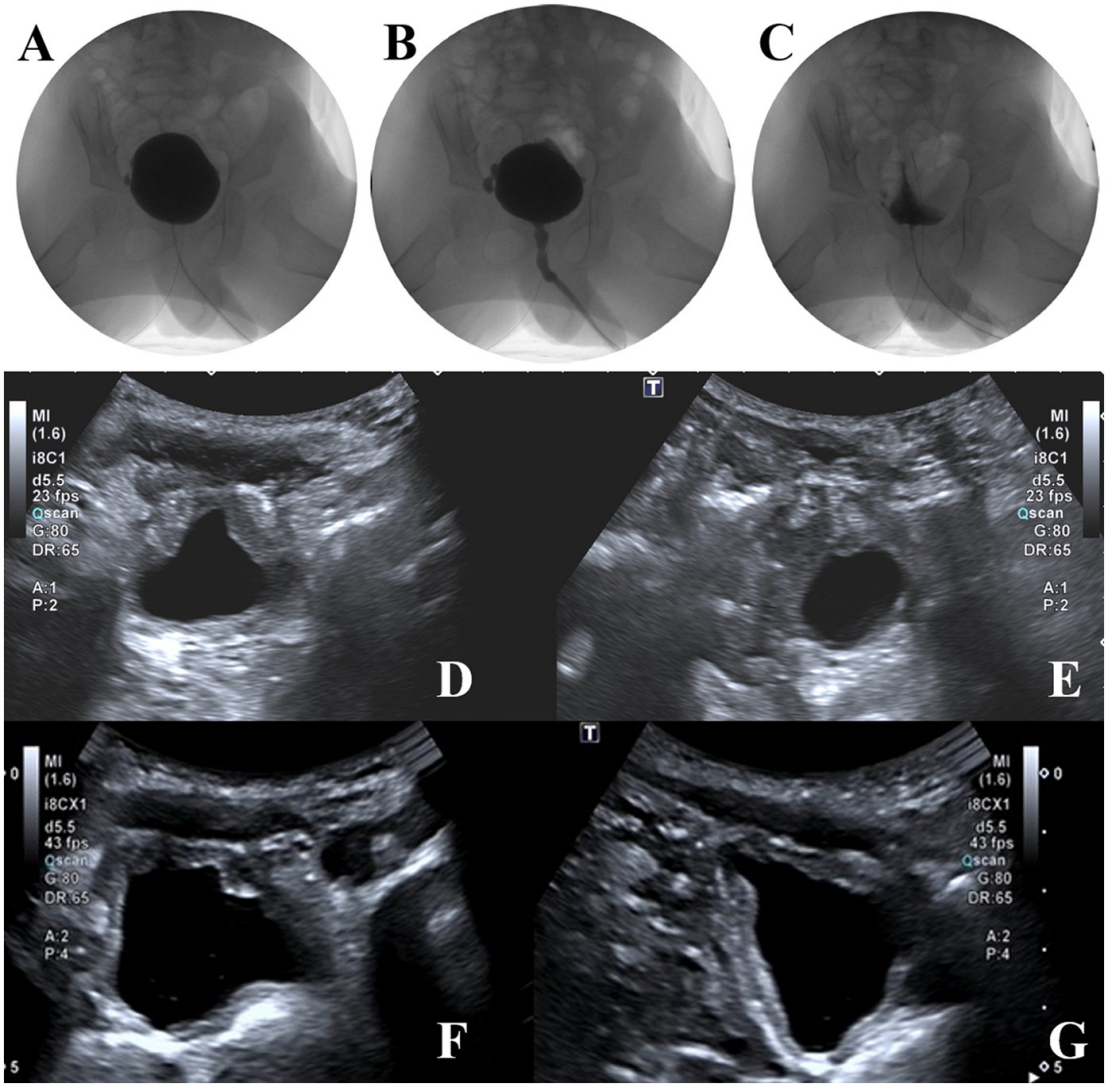
Postoperative video urodynamic study images showing the bladder filling phase **(A)**, voiding phase **(B)**, and post-void phase **(C)** follow-up ultrasound images demonstrating no new diverticula and improved bladder morphology **(D–G)**.

At the one-year follow-up, repeat video urodynamic testing demonstrated marked improvement in bladder function. Bladder compliance increased from 13 to 21 ml/cm H₂O, and post-void residual urine volume decreased from 54 ml to 5 ml. The detrusor exhibited normal contractility, and the detrusor-sphincter coordination was characterized as Type II dyssynergia. The end-filling detrusor pressure remained stable at 5 cm H₂O. In addition, ultrasound assessments performed at one and three months postoperatively showed residual urine volumes of 16 ml and 8 ml, respectively, indicating progressive improvement in bladder emptying. The images during the bladder filling period, the urination period and after urination are shown in Figures 4A–C. Imaging studies confirmed the absence of newly formed diverticula. The patient maintained satisfactory urinary function with no recurrence of abdominal distension, urinary tract infection or urinary retention, nor was there vesicoureteral reflux (see Figures 4D–G).

Given the progressive nature of Menkes syndrome, the patient will continue to undergo long-term urological monitoring, including annual urodynamic evaluations, regular renal and bladder ultrasonography, and ongoing clinical assessment for urinary symptoms. This extended follow-up aims to promptly identify any recurrence of diverticula or new-onset lower urinary tract dysfunction.

## Discussion

This case report describes a child with Menkes syndrome complicated by multiple bladder diverticula and diverticular rupture. The use of imaging and urodynamic assessments guided surgical treatment and follow-up, yielding favorable clinical outcomes. This case provides valuable insights into the management of rare urological complications in Menkes syndrome and underscores the importance of comprehensive evaluation (1).

### The relationship between Menkes syndrome and bladder diverticula

Menkes syndrome is a disorder of copper metabolism caused by variants in the *ATP7A* gene. The pathological mechanism involves a decrease in the activity of copper-dependent enzymes, such as lysyl oxidase, which leads to abnormal cross-linking of collagen fibers in connective tissues (4). This abnormality weakens the bladder wall structure, making it prone to diverticula formation (5). The discovery of multiple bladder diverticula in this case suggests that patients with Menkes syndrome may have an inherent vulnerability in the urological system, highlighting the need for early screening for urological complications (8).

### The role of surgical excision in the treatment of bladder diverticula

Currently, there are no standardized guidelines for the treatment of bladder diverticula associated with Menkes syndrome. In this case, the rupture of a diverticulum led to acute abdominal effusion, suggesting that the presence of diverticula may result in severe complications. While some literature recommends conservative treatment for small or asymptomatic diverticula, surgical excision may be a more effective option for diverticula that are associated with functional impairment or rupture risk (3, 9). In this study, excision of multiple bladder diverticula significantly improved the patient's voiding function, and follow-up confirmed the absence of recurrence or new diverticula formation. These findings support the clinical value of surgical intervention in severe cases and emphasize that surgical strategies should be tailored based on individual patient conditions and comprehensive evaluation results (6, 10).

### The role of imaging and urodynamics in diagnosis and follow-up

Imaging and urodynamic assessments played a key role in the diagnosis, treatment, and follow-up of this case. Preoperative urodynamic studies not only localized the bladder diverticula but also evaluated their impact on bladder function, providing the basis for surgical intervention (11). Postoperative follow-up urodynamics showed improvements in bladder capacity and emptying function, objectively assessing the efficacy of the surgery and monitoring potential recurrence. Given the complex pathological features of Menkes syndrome, imaging and urodynamics, as non-invasive and accurate tools, are invaluable for long-term management of urological complications in these patients (6, 12, 13).

### The significance of a multidisciplinary treatment strategy

The treatment goal for Menkes syndrome extends beyond improving the patient's quality of life to actively managing its multisystem complications. This case demonstrates how a multidisciplinary team approach can formulate an individualized treatment plan that includes imaging, surgery, and postoperative follow-up, effectively reducing the patient's disease burden. While current treatment for Menkes syndrome primarily focuses on copper supplementation and symptom management (5), this study emphasizes the importance of early identification and intervention for comorbidities, particularly rare but potentially life-threatening urological complications such as bladder diverticula.

### Limitations and future directions

The limitations of this study include the small sample size due to the single case report, which may not fully represent the clinical characteristics of bladder diverticula in Menkes syndrome. Additionally, the long-term outcomes of surgical excision require further follow-up.

Despite these limitations, this case offers valuable clinical insights. It underscores the importance of considering bladder diverticula and potential rupture in Menkes patients presenting with unexplained abdominal symptoms or recurrent urinary tract issues. Early integration of imaging and urodynamic studies may facilitate timely diagnosis and prevent serious complications. In particular, the observed functional improvement following surgical resection highlights the potential benefit of proactive surgical intervention in selected patients. Future multicenter collaborations and large-sample studies are warranted to establish standardized screening protocols and optimize management strategies. Additionally, the long-term utility of imaging and urodynamics in disease monitoring should be further clarified.

## Data Availability

The original contributions presented in the study are included in the article/Supplementary Material, further inquiries can be directed to the corresponding authors.
